# Association of blood parameters and ratios with aseptic loosening after total knee arthroplasty: A retrospective cohort study

**DOI:** 10.1097/MD.0000000000050825

**Published:** 2026-09-18

**Authors:** Mehmet Kurt, Selcuk Yilmaz, Turan Cihan Dulgeroglu, Emre Mucahit Kartal

**Affiliations:** a Department of Orthopedics and Traumatology, Kütahya Health Sciences University, Kütahya, Türkiye; b Department of Orthopedics and Traumatology, Akdeniz University, Antalya, Türkiye; c Department of Orthopedics and Traumatology, Antalya City Hospital, Antalya, Türkiye.

**Keywords:** knee prosthesis, knee surgery, loosening

## Abstract

This study aimed to evaluate the association of blood parameters and ratios with knee prosthesis loosening in patients after knee arthroplasty. A total of 221 patient files having knee prosthesis attempting to our clinic between 2018 and 2022 were retrospectively examined. Patients were divided into 2 groups as loosening (n = 108; 48.9%) and not loosening (n = 113; 51.1%) groups. Monocyte/high-density lipoprotein (HDL) ratio monocyte-to-HDL ratio (MHR), Neutrophil/lymphocyte ratio, Albumin/ C-reactive protein (CRP) ratio, albumin-to-C-reactive protein ratio (ACR), Eosinophile-to- lymphocyte ratio, mean platelet volume /PLT ratio mean platelet volume-to-platelet ratio, Monocyte -to-lymphocyte ratio, Platelet/lymphocyte ratio (PLR) and Hemoglobin/Platelet ratio (HPR) parameters of patients were evaluated. HDL, albumin, hemoglobin, ACR, PLR and HPR levels were significantly higher in loosening patients (*P* < .05). CRP, neutrophile, lymphocyte, monocyte and MHR means were significantly higher in no-loosening patients (*P* < .05). Loosening was significantly correlated with gender (*R* = 0.141; *P* < .05), HDL (*R* = 0.474; *P* < .01), albumin (*R* = 0.349; *P* < .01), CRP (r = −0.466; *P* < .01), neutrophile (r = −0.167; *P* < .05), lymphocyte (r = −0.240; *P* < .01), monocyte (r = −0.380; *P* < .01), hemoglobin (*R* = 0.254; *P* < .01), MHR (r = −0.513; *P* < .01), ACR (*R* = 0.496; *P* < .01), PLR (*R* = 0.142; *P* < .05) and HPR (*R* = 0.204; *P* < .01). Binary logistic regression analysis showed that MHR (B = −0.415; *P* < .01), ACR (B = 0.048; *P* < .01), and HPR (B = 30.640; *P* < .05) were significantly associated with knee prosthesis loosening at the multivariate level. ROC analysis demonstrated statistically significant discriminative performance for MHR, area under the curve ([AUC] = 0.798; *P* < .01), ACR (AUC = 0.786; *P* < .01), and HPR (AUC = 0.618; *P* < .01). For MHR 6.2481 cutoff level, sensitivity was 72.2% and specificity was 10.6%. For ACR 8.6380 cutoff level, sensitivity was 84.3% and specificity was 59.3%. For HPR 0.0506 cutoff level, sensitivity was 63.9% and specificity was 54.0%. MHR, ACR, and HPR were significantly associated with aseptic loosening after total knee arthroplasty and may be considered potential associated markers rather than definitive predictors.

## 1. Introduction

Knee osteoarthritis (OA) is a chronic degenerative condition characterized by the gradual degradation of articular cartilage within the knee joint, often leading to persistent pain and functional impairment.^[1]^ Total knee arthroplasty (TKA) has become a cornerstone in the management of advanced knee OA, particularly in patients with significant pain and functional limitations. The demand for TKA is increasing globally, largely due to rising life expectancy and an aging population.^[2]^ TKA is the most commonly performed surgical procedure for severe medial knee osteoarthritis, offering relief from pain and improvement in joint function.^[3]^

However, despite its widespread success, postoperative complications remain a concern. Within the first 90 days following primary TKA, approximately 4% of patients experience an adverse event, while 8% report dissatisfaction with the outcome.^[4]^ One of the most challenging complications is pain, which may occur without evidence of prosthetic loosening, as well as pain related to aseptic loosening. Both of these factors significantly diminish the quality of life for patients following TKA.^[5]^ Moreover, research indicates that TKA performed in the setting of periprosthetic joint infection (PJI) is associated with higher rates of aseptic loosening, underscoring the importance of early diagnosis and appropriate management in these patients.^[2]^

The early identification of prosthetic loosening is crucial, as it can lead to more severe complications if left untreated. Loosening not only compromises joint function and mobility, but it also contributes to significant patient morbidity, highlighting the need for close monitoring and timely intervention following TKA.^[6]^ Furthermore, the occurrence of aseptic loosening is associated with a range of complications, including prosthetic failure and the need for revision surgery, which underscores the complexity of managing this condition.^[7,8]^ Therefore, continuous research and refinement of diagnostic and treatment protocols are essential to improving patient outcomes and minimizing long-term complications following TKA.

Several studies have suggested that genetic factors may play a significant role in the development of aseptic loosening following TKA, indicating that certain genetic predispositions could influence the risk of this complication.^[9,10]^ In addition to genetic factors, demographic characteristics, including age, sex, and body mass index, have also been identified as potential risk factors for the occurrence of complications after TKA.^[11–13]^ Furthermore, the material composition and structural design of the prosthesis have been highlighted in the literature as important determinants of the likelihood of aseptic loosening, with certain materials and designs being associated with higher failure rates.^[13–16]^

Despite the recognition of these risk factors, there remains a lack of consensus on whether other laboratory markers associated with aseptic loosening exist. The complications associated with this condition can lead to a poor clinical prognosis, making the identification of potential associated markers clinically relevant. Early detection and intervention to prevent the progression of loosening are paramount for improving patient outcomes. As such, it is essential to not only identify risk factors but also to understand their relative impact on the development of aseptic loosening.

While there is a growing body of research addressing the complications related to knee replacement and aseptic loosening, there is a notable gap in studies focused on laboratory markers associated with knee prosthesis loosening. Given the importance of early intervention, the current study aims to evaluate the association of blood parameters and ratios with knee prosthesis loosening in patients following TKA. Identifying reliable biomarkers could significantly improve the management and outcomes of these patients by facilitating early detection and timely treatment interventions.

## 2. Methods

### 2.1. Model of the research

The study was designed as a retrospective cohort study. The medical records of patients who underwent primary total knee arthroplasty between 2018 and 2022 were retrospectively reviewed, and patients were classified according to whether they subsequently developed aseptic loosening during follow-up.

### 2.2. Sample size

A total of 221 patient files from individuals who underwent TKA at Kütahya Health Sciences University Hospital between 2018 and 2022 were retrospectively analyzed. The patients were categorized into 2 groups: the loosening group (n = 108; 48.9%) and the non-loosening group (n = 113; 51.1%). The inclusion criteria are as follows:

- Patients who had undergone TKA with a minimum of 6 months of prosthetic follow-up data.
- Patients with complete medical records, including all relevant parameters.
- Patients without chronic diseases that could potentially impact the research outcomes.
- Patients who were not using medications that could adversely affect the results of the study.

Aseptic loosening was defined as clinical symptoms consistent with mechanical loosening accompanied by progressive radiolucent lines, component migration/subsidence, a change in component position, or progressive periprosthetic osteolysis on serial radiographs, in the absence of evidence of periprosthetic joint infection. Patients meeting these criteria were classified as the aseptic loosening group, whereas patients without clinical or radiological evidence of loosening during follow-up were classified as the non-loosening group. The diagnosis was determined by an orthopedic surgeon experienced in arthroplasty based on the clinical and radiological findings.

### 2.3. Research parameters

The following parameters were calculated for the patients:monocyte-to-HDL ratio(MHR), Neutrophil/lymphocyte ratio, Albumin/C-reactive protein (CRP) ratio, albumin-to-C-reactive protein ratio (ACR), Eosinophil -to-lymphocyte ratio, mean platelet volume /PLT ratio mean platelet volume-to-platelet ratio, Monocyte/lymphocyte ratio, Platelet/lymphocyte ratio (PLR), and Hemoglobin-to-Platelet ratio (HPR). Preoperative laboratory parameters obtained before primary total knee arthroplasty were retrospectively retrieved from the patients’ medical records. The same preoperative time point was used for both patients who subsequently developed aseptic loosening and those who remained free of loosening during follow-up.

### 2.4. Statistical methods

Gender distribution was described with frequencies, whereas scale parameters were described with means and standard deviations. Fisher’s exact test was used for gender differences between patient groups. Normality of scale parameters were analyzed with Kolmogorov Smirnov test. For non-normally distributed parameters, Mann–Whitney *U* test was used. For normally distributed parameter differences, Independent Samples *t* test was used. Spearman’s rho correlation and Binary Logistic Regression analysis were used for relationship design due to linearization deviations in nonparametric models.^[17]^ Variables demonstrating statistically significant associations with prosthesis loosening in the preliminary analyses were considered for the multivariable model. Given the primary focus of the study on blood-derived ratios, the significantly associated ratio-based parameters (MHR, ACR, PLR, and HPR) were entered into the binary logistic regression model, while gender was included as a demographic variable because it was significantly associated with loosening. To reduce redundancy and potential multicollinearity, the individual laboratory components used to calculate these ratios were not entered simultaneously with their corresponding ratio variables. ROC analysis was used for diagnostic values of significant parameters. SPSS 17.0 for Windows was used for analysis at 95% confidence and a 0.05 significance level.

### 2.5. Ethical considerations

Ethics committee approval was obtained from the Kütahya Health Sciences University Rectorate Non-Interventional Clinical Research Ethics Committee (Decision No. 2022/10–13; Date: 12 October, 2022). The requirement for informed consent was waived by the ethics committee due to the retrospective nature of the study.

## 3. Results

The proportion of female patients in the loosening group was significantly lower, while the proportion of male patients was higher (*P* < .05). Additionally, levels of HDL, albumin, hemoglobin, ACR, PLR, and HPR were significantly higher in the loosening group (*P* < .05). In contrast, the mean values of neutrophils, lymphocytes, monocytes, and the MHR were significantly higher in the non-loosening group (*P* < .05) (Table 1).

**Table 1:** Gender, age, and clinical parameters with indicators for patient groups and difference analysis results.

	Aseptic loosening		*P* value
	No (n = 113; 51.1%)	Yes (n = 108; 48.9%)	
Gender, n (%)			.027^a^
Female	99 (87.6)	83 (76.9)	
Male	14 (12.4)	25 (23.1)	
Age	69.87 ± 5.83	70.44 ± 0.50	.153^b^
HDL	46.98 ± 7.96	54.98 ± 7.53	.000^a^
Albumin	3.67 ± 0.45	4.03 ± 0.41	.000^b^
CRP	7.80 ± 7.72	6.88 ± 35.34	.000^b^
Neutrophile	4.98 ± 1.56	4.45 ± 1.42	.013^b^
Lymphocyte	2.06 ± 0.77	1.75 ± 0.58	.000^b^
Monocyte	0.52 ± 0.15	0.41 ± 0.09	.000^b^
Eosinophile	0.15 ± 0.12	0.13 ± 0.07	.782^b^
MPV	9.50 ± 1.02	9.24 ± 0.95	.069^b^
Hemoglobin	12.71 ± 1.44	13.42 ± 1.25	.000^b^
Platelet	260.54 ± 70.01	243.82 ± 61.85	.108^b^
MHR	11.45 ± 4.17	7.67 ± 2.11	.000^b^
NLR	2.72 ± 1.28	2.97 ± 2.10	.741^b^
ACR	10.86 ± 15.35	23.61 ± 34.10	.000^b^
ELN	0.08 ± 0.07	0.08 ± 0.06	.072^b^
MPR	0.04 ± 0.01	0.04 ± 0.01	.333^b^
MLR	0.28 ± 0.13	0.26 ± 0.10	.391^b^
PLR	140.39 ± 59.84	154.62 ± 66.43	.036^b^
HPR	0.05 ± 0.02	0.06 ± 0.02	.002^b^

ACR = albumin-to-C-reactive protein ratio, CRP = C-reactive protein, ELN = eosinophil-to-lymphocyte ratio, HDL = high-density lipoprotein, HPR = hemoglobin-to-platelet ratio, MHR = monocyte-to-high-density lipoprotein ratio, MLR = monocyte-to-lymphocyte ratio, MPR = mean platelet volume-to-platelet ratio, MPV = mean platelet volume, n = number of patients, NLR = neutrophil-to-lymphocyte ratio, PLR = platelet-to-lymphocyte ratio.

a . Fisher’s Exact Test.

b . Mann Whitney *U* Test.

c . Independent Samples *t* test.

The analysis revealed significant correlations between the loosening group and various parameters, including gender (*R* = 0.141; *P* < .05), HDL (*R* = 0.474; *P* < .01), albumin (*R* = 0.349; *P* < .01), CRP (*r* = −0.466; *P* < .01), neutrophil count (*r* = −0.167; *P* < .05), lymphocyte count (*r* = −0.240; *P* < .01), monocyte count (*r* = −0.380; *P* < .01), hemoglobin levels (*R* = 0.254; *P* < .01), MHR (*r* = −0.513; *P* < .01), ACR (*R* = 0.496; *P* < .01), PLR (*R* = 0.142; *P* < .05), and HPR (*R* = 0.204; *P* < .01), as detailed in Table 2.

**Table 2:** Spearman’s rho correlation between loosening and research parameters.

Aseptic loosening	*r*	*p*
Gender	.141*	.036
Age	.096	.153
HDL	.474**	.000
Albumin	.349**	.000
CRP	−.466**	.000
Neutrophile	−.167*	.013
Lymphocyte	−.240**	.000
Monocyte	−.380**	.000
Eosinophile	.019	.782
MPV	−.123	.069
Hemoglobin	.254**	.000
Platelet	−.108	.108
MHR	−.513**	.000
NLR	.022	.742
ACR	.496**	.000
ELN	.121	.073
MPR	.065	.334
MLR	−.058	.392
PLR	.142*	0.035
HPR	.204**	.002

ACR = albumin-to-C-reactive protein ratio, CRP = C-reactive protein, ELN = eosinophil-to-lymphocyte ratio, HDL = high-density lipoprotein, HPR = hemoglobin-to-platelet ratio, MHR = monocyte-to-high-density lipoprotein ratio, MLR = monocyte-to-lymphocyte ratio, MPR = mean platelet volume-to-platelet ratio, MPV = mean platelet volume, NLR = neutrophil-to-lymphocyte ratio, PLR = platelet-to-lymphocyte ratio, r = Spearman’s rho correlation coefficient.

* *P*<.05.

** *P*<.01.

The binary logistic regression analysis indicated that MHR (B = -0.415; *P* < .01), ACR (B = 0.048; *P* < .01), and HPR (B = 30.640; *P* < .05) were significantly associated with aseptic loosening at the multivariate level (Table 3).

**Table 3:** Binary logistic regression analysis for loosening and related parameters.

	B	S.E.	Wald	*P*	OR	95% CI for OR	
						Lower	Upper
Gender	−,555	,468	1407	,236	,574	,230	1436
MHR	−,415	,070	35,038	,000	,661	,576	,758
ACR	,048	,014	11,482	,001	1049	1020	1078
PLR	,005	,003	2315	,128	1005	,999	1011
HPR	30,640	12,403	6103	,013	20.26,305	561,773	73.07,000
Constant	1061	1307	,659	,417	2889		

ACR = albumin-to-C-reactive protein ratio, C.I = confidence interval, HPR = hemoglobin-to-platelet ratio, MHR = monocyte-to-high-density lipoprotein ratio, OR = odds ratio, PLR = platelet-to-lymphocyte ratio, S.E = standard error.

**Figure 1.**
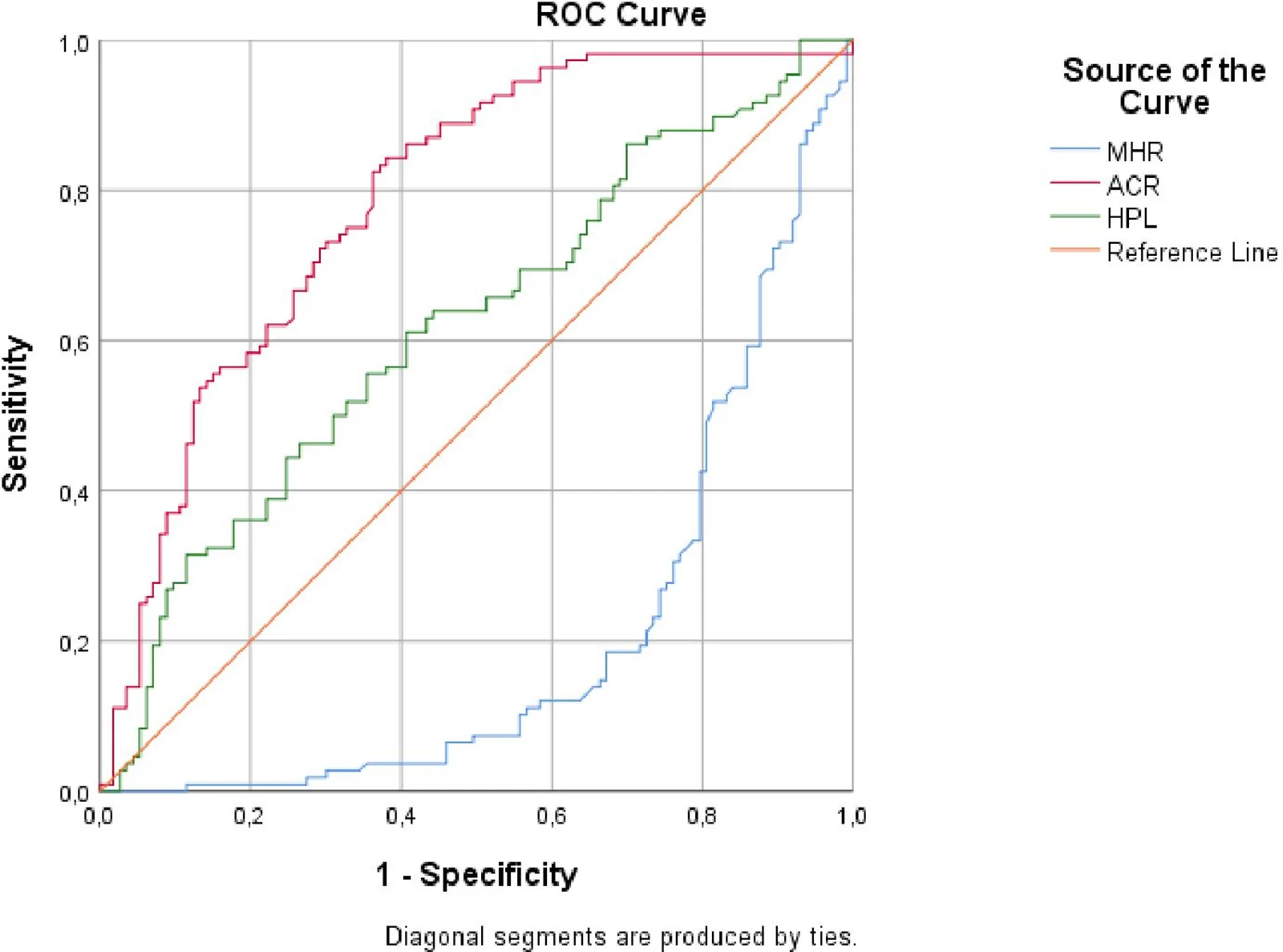
Receiver operating characteristic (ROC) curves for MHR, ACR, and HPR for the discrimination of aseptic loosening after total knee arthroplasty. ACR = albumin-to-C-reactive protein ratio, HPR = hemoglobin-to-platelet ratio, ROC = receiver operating characteristic.

ROC analysis demonstrated statistically significant discriminative performance for MHR, area under the curve ([AUC] = 0.798; P < .01), ACR (AUC = 0.786; P < .01), and HPR (AUC = 0.618; P < .01). For the MHR 6.2481 cutoff level, sensitivity was 72.2% and specificity was 10.6%. For the ACR 8.6380 cutoff level, sensitivity was 84.3% and specificity was 59.3%. For the HPR 0.0506 cutoff level, sensitivity was 63.9% and specificity was 54.0% (Fig. 1).

## 4. Discussion

Aseptic loosening of the knee prosthesis is an undesirable complication following knee surgeries, which increases with age and negatively impacts the quality of life.^[18–20]^ In order to mitigate the negative effects of aseptic loosening on the health status of patients and to manage the treatment process effectively, it is essential to have a comprehensive understanding of its causes, mechanisms, and consequences.^[21,22]^ Therefore, identifying clinical or laboratory markers associated with aseptic loosening may be useful for further investigation and clinical assessment.

Although aseptic loosening following TKA has been the subject of several studies in the literature,^[23–25]^ there remains a general lack of research establishing a direct relationship between aseptic loosening and demographic characteristics. In the limited studies available, older age has been identified as a significant demographic risk factor, with a more pronounced effect observed in women. In our study, although the average age of patients in the aseptic loosening group was higher than that of the non-loosening group, no statistically significant difference was found between the ages of the 2 groups. Notably, the average age in both groups was above 69 years. Regarding gender, the loosening group had a significantly higher proportion of males compared to the non-loosening group. However, the proportion of females was substantially higher in both groups. This finding suggests that TKA is performed more frequently in women. The reason for the higher proportion of males in the loosening group cannot be determined from the available retrospective data and may be influenced by unmeasured clinical or patient-related factors.

Regarding the indicators, clinical structure, and blood values that changed with age, notable differences were observed in both groups. However, based on the research results, the differences in MHR, ACR, PLR, and HPR values between the groups were statistically significant. The correlation analysis also showed significant differences in these variables between the groups. Although multiple factors were associated with loosening in the individual analyses, only MHR, ACR, and HPR remained significantly associated with aseptic loosening in the multivariate analysis. ROC analysis further demonstrated statistically significant discriminative performance for these 3 markers. These findings should be interpreted as associations observed in a retrospective analysis rather than as evidence of definitive prospective prediction.

### 4.1. Limitations of the study

The primary limitation of this study is that it was conducted on a very specific sample group, namely, a geriatric population aged over 65. In general, geriatric groups may present with multiple chronic diseases, medication use, comorbid conditions, or other health issues. Although patients with chronic diseases or medication use that could potentially affect the study outcomes were excluded from the evaluation, there may still be individuals with undiagnosed or asymptomatic health conditions. Therefore, this research could be expanded by including a larger sample size and patients who undergo more comprehensive health assessments. In addition, the retrospective design may have introduced selection bias and residual confounding. Potentially relevant factors such as body mass index, implant-related characteristics, and fixation technique were not comprehensively accounted for and may have influenced the observed associations.

Another significant limitation is the limited availability of data in both the literature and practical applications. While this may position the research as a pioneering study in the field, semi-qualitative and quantitative data, such as additional demographic information, details related to daily and social life, and quality of life measures, were not adequately captured. Consequently, the level of information available in the patients’ files represents another important limitation of the study.

### 4.2. Contributions of the research to the literature

The most significant contribution of this study to the literature is the evaluation of blood parameters as potential markers associated with TKA loosening rather than definitive predictors. In this regard, the research can be considered one of the pioneering studies in the field, making a notable contribution to the existing literature.

Another important contribution of the research to the literature comes from its pragmatic structure and research design for clinical applications. Today, many new studies on geriatrics are being conducted and published all over the world. In this respect, the research is also important in terms of making a positive contribution to a very specific group within the geriatric group.

## 5. Conclusion

The results of this retrospective study indicate that MHR, ACR, and HPR are significantly associated with aseptic loosening after TKA and demonstrate discriminative performance. These parameters should be considered potential associated markers rather than definitive predictors, and prospective validation is required before their use for individual risk prediction.

## Author contributions

**Data curation:** Mehmet Kurt, Turan Cihan Dulgeroglu.

**Funding acquisition:** Mehmet Kurt, Selcuk Yilmaz.

**Project administration:** Mehmet Kurt, Emre Mucahit Kartal.

**Validation:** Selcuk Yilmaz, Turan Cihan Dulgeroglu.

**Formal analysis:** Turan Cihan Dulgeroglu.

**Supervision:** Emre Mucahit Kartal.

**Writing – original draft:** Mehmet Kurt.

**Writing – review & editing:** Selcuk Yilmaz, Emre Mucahit Kartal.
